# Supplementary material for: The Association of Attending Physicians’ Publications and Patients’ Readmission Rates: Evidence from Tertiary Hospitals in China Using a Retrospective Data Analysis
Source: Int J Environ Res Public Health. 2022 Aug 8;19(15):9760. doi: 10.3390/ijerph19159760 (PMC9368559; doi:10.3390/ijerph19159760)
Supplement: Supplementary file 1 [file ijerph-19-09760-s001.zip › ijerph-1827950-supplementary.pdf]

Supplementary Table

Table S1: Physicians' First-Author Publications

	Internist %	Surgeon %
Publication Type	Physician Level	
Any publication	37.77	41.22
Average impact factor 3+	17.65	16.10
Average impact factor 5+	4.34	3.70
Any clinical study	23.04	24.62
Average impact factor 3+	4.79	3.25
Average impact factor 5+	1.25	0.73
Any basic science study	30.84	34.04
Average impact factor 3+	11.82	12.00
Average impact factor 5+	3.12	3.20
N	4,011	1,783
	Visit Level	
Any publication	34.05	36.56
Average impact factor 3+	15.09	12.98
Average impact factor 5+	4.06	3.11
Any clinical study	20.64	22.45
Average impact factor 3+	3.97	2.30
Average impact factor 5+	1.08	0.71
Any basic science study	28.17	29.39
Average impact factor 3+	10.06	10.19
Average impact factor 5+	3.24	2.45
N	84,954	27,011

Note. Physician-level data provide information on the characteristics of the physician. However, because we conducted the analysis at the visit level, we also provide information on characteristics of the physician at the visit level.

Table S2: Descriptive Statistics-Patient Characteristics

Characteristic	Internist (n=4011)	Surgeon (n=1783)
Hospitalizations, n	84,954	27,011
Female, %	52.53	50.09
Age, mean (sd)	59.33 (16.91)	56.47 (17.37)
Length of stay (day) , mean (sd)	9.06 (6.81)	7.75 (6.91)
Number of outpatient visits, mean (sd)	23.67 (25.85)	21.86 (25.59)
Residence, %		
Rural	13.05	16.26
Urban	85.44	82.06
Other	1.53	1.68
Position, %		
Worker	60.39	61.77
Enterprise manager	16.06	15.89
Freelancer	17.90	17.42
Other	5.17	5.65
Number of chronic diseases, %		
None	39.40	55.17
One	21.73	19.72
Multiple	38.87	25.11

Note. Standard deviations are shown in parentheses.

Table S3: Association Between Physicians' First-Author Publication and Patients' 30-Day Readmission Rates (Using Non-logistic Regression)

Publication Type	Model 1 Adjusted Risk Difference	Model 1 *p*	Model 2 Adjusted Risk Difference	Model 2 *p*
**Panel A. Internist**				
Any publication	-0.005 [-0.015, 0.005]	.302	-0.001 [-0.010, 0.008]	.783
Average impact factor 3+	-0.013** [-0.024, -0.002]	.020	-0.008 [-0.019, 0.002]	.125
Average impact factor 5+	-0.025*** [-0.042, -0.007]	.006	-0.018** [-0.035, -0.002]	.028
Any clinical study	0.000 [-0.012, 0.012]	.959	0.001 [-0.010, 0.012]	.819
Average impact factor 3+	-0.032*** [-0.050, -0.014]	.001	-0.023** [-0.042, -0.005]	.012
Average impact factor 5+	-0.043*** [-0.071, -0.015]	.002	-0.038*** [-0.067, -0.010]	.008
Any basic science study	-0.008 [-0.019, 0.002]	.108	-0.005 [-0.014, 0.004]	.259
Average impact factor 3+	0.001 [-0.015, 0.017]	.897	0.001 [-0.013, 0.014]	.918
Average impact factor 5+	-0.009 [-0.031, 0.012]	.391	-0.004 [-0.024, 0.015]	.663
**Panel B. Surgeon**				
Any publication	0.007 [-0.007, 0.021]	.328	0.012* [-0.001, 0.026]	.078
Average impact factor 3+	0.008 [-0.010, 0.026]	.405	0.008 [-0.009, 0.026]	.359
Average impact factor 5+	-0.016 [-0.040, 0.008]	.186	-0.010 [-0.035, 0.014]	.405
Any clinical study	-0.002 [-0.021, 0.017]	.868	0.004 [-0.013, 0.021]	.632
Average impact factor 3+	-0.048*** [-0.075, -0.020]	.001	-0.039*** [-0.067, -0.011]	.006
Average impact factor 5+	-0.084*** [-0.134, -0.034]	.001	-0.077*** [-0.128, -0.027]	.003
Any basic science study	0.005 [-0.007, 0.018]	.427	0.009 [-0.004, 0.021]	.166
Average impact factor 3+	0.023** [0.004, 0.043]	.017	0.023** [0.003, 0.042]	.025
Average impact factor 5+	-0.001 [-0.024, 0.022]	.946	0.003 [-0.022, 0.027]	.839

Note. Standard deviations are shown in parentheses, and 95% confidence intervals are shown in brackets. Model 1 adjusts for patient characteristics, and Model 2 further adjusts for hospital fixed effects.
*p < .10. **p < .05. ***p < .01.

Table S4: Association Between Physicians' First-Author Publications and Patients' 30-Day Readmission Rates (Without Control Variables)

	Model 1		Model 2	
Publication Type	Adjusted Odds Ratio	*p*	Adjusted Odds Ratio	*p*
	Panel A. Internist			
Any publication	0.952 [0.886, 1.023]	.178	0.989 [0.928, 1.054]	.730
Average impact factor 3+	0.895*** [0.825, 0.970]	.007	0.953 [0.882, 1.030]	.223
Average impact factor 5+	0.824*** [0.721, 0.943]	.005	0.888* [0.784, 1.006]	.061
Any clinical study	0.988 [0.905, 1.080]	.797	1.014 [0.939, 1.095]	.724
Average impact factor 3+	0.761*** [0.663, 0.873]	< .001	0.850** [0.739, 0.978]	.023
Average impact factor 5+	0.718*** [0.581, 0.888]	.002	0.781** [0.633, 0.962]	.020
Any basic science study	0.934* [0.866, 1.008]	.078	0.960 [0.899, 1.025]	.223
Average impact factor 3+	0.994 [0.883, 1.119]	.918	1.008 [0.916, 1.108]	.875
Average impact factor 5+	0.928 [0.794, 1.086]	.353	0.967 [0.830, 1.127]	.671
	Panel B. Surgeon			
Any publication	1.051 [0.934, 1.184]	.407	1.105* [0.998, 1.224]	.054
Average impact factor 3+	1.067 [0.917, 1.241]	.403	1.086 [0.945, 1.248]	.245
Average impact factor 5+	0.852 [0.661, 1.098]	.214	0.936 [0.735, 1.190]	.588
Any clinical study	0.980 [0.830, 1.156]	.810	1.024 [0.902, 1.163]	.710
Average impact factor 3+	0.619*** [0.455, 0.842]	.002	0.693** [0.516, 0.932]	.015
Average impact factor 5+	0.472*** [0.272, 0.820]	.008	0.590* [0.332, 1.047]	.071
Any basic science study	1.048 [0.939, 1.171]	.403	1.096* [0.992, 1.211]	.073
Average impact factor 3+	1.231*** [1.052, 1.440]	.010	1.223** [1.048, 1.429]	.011
Average impact factor 5+	0.990 [0.782, 1.253]	.930	0.995 [0.774, 1.279]	.967

Note. Standard deviations are shown in parentheses, and 95% confidence intervals are shown in brackets. Model 1 didn't include any control, and Model 2 adjusts for hospital fixed effects.

**p* < .10. ***p* < .05. ****p* < .01.

Table S5: Association Between Physicians' First-Author Publications and Patients' 60-Day Readmission Rates

	Publication		Model 1		Model 2	
Publication Type	Yes	No	Adjusted Odds Ratio	*p*	Adjusted Odds Ratio	*p*
			Panel A. Internist			
Any publication	0.23	0.25	0.962	.212	0.985	.615
	(0.42)	(0.43)	[0.905, 1.022]		[0.931, 1.043]	
Average impact factor 3+	0.22	0.25	0.924**	.033	0.952	.161
	(0.42)	(0.43)	[0.859, 0.994]		[0.889, 1.020]	
Average impact factor 5+	0.22	0.24	0.901*	.098	0.943	.301
	(0.41)	(0.43)	[0.797, 1.019]		[0.843, 1.054]	
Any clinical study	0.24	0.25	0.990	.798	0.997	.930
	(0.42)	(0.43)	[0.919, 1.067]		[0.931, 1.068]	
Average impact factor 3+	0.19	0.25	0.818***	.002	0.879**	.034
	(0.39)	(0.43)	[0.720, 0.929]		[0.780, 0.990]	
Average impact factor 5+	0.19	0.24	0.746***	.002	0.791**	.011
	(0.39)	(0.43)	[0.618, 0.901]		[0.660, 0.949]	
Any basic science study	0.22	0.25	0.939*	.055	0.955	.125
	(0.42)	(0.43)	[0.880, 1.001]		[0.900, 1.013]	
Average impact factor 3+	0.25	0.24	1.014	.791	1.004	.923
	(0.43)	(0.43)	[0.917, 1.120]		[0.920, 1.096]	
Average impact factor 5+	0.23	0.24	1.019	.805	1.037	.606
	(0.42)	(0.43)	[0.879, 1.181]		[0.903, 1.192]	
			Panel B. Surgeon			
Any publication	0.18	0.17	1.045	.373	1.102**	.046
	(0.38)	(0.37)	[0.948, 1.153]		[1.002, 1.213]	
Average impact factor 3+	0.18	0.17	1.076	.339	1.094	.188
	(0.38)	(0.37)	[0.926, 1.251]		[0.957, 1.250]	
Average impact factor 5+	0.15	0.17	0.889	.327	0.956	.700
	(0.35)	(0.38)	[0.703, 1.124]		[0.763, 1.199]	
Any clinical study	0.17	0.17	0.998	.971	1.053	.348
	(0.38)	(0.38)	[0.886, 1.124]		[0.945, 1.174]	
Average impact factor 3+	0.14	0.17	0.715**	.016	0.797*	.095
	(0.35)	(0.38)	[0.545, 0.939]		[0.611, 1.040]	
Average impact factor 5+	0.13	0.17	0.447***	.005	0.523**	.03
	(0.34)	(0.38)	[0.256, 0.779]		[0.291, 0.940]	
Any basic science study	0.17	0.17	1.057	.255	1.104**	.036
	(0.38)	(0.38)	[0.960, 1.164]		[1.007, 1.211]	
Average impact factor 3+	0.19	0.17	1.203**	.027	1.197**	.017
	(0.39)	(0.37)	[1.022, 1.415]		[1.033, 1.387]	
Average impact factor 5+	0.15	0.17	1.014	.889	1.035	.751
	(0.35)	(0.38)	[0.833, 1.235]		[0.836, 1.281]	

Note. Standard deviations are shown in parentheses, and 95% confidence intervals are shown in brackets. Model 1 adjusts for patient characteristics, and Model 2 further adjusts for hospital fixed effects.
$*p < .10$. $**p < .05$. $***p < .01$.

Table S6: Association Between Physicians' First-Author Publications and Patients' 30-Day Readmission Rates (Using Additional Controls/Mechanisms)

	Model 1		Model 2		Model 3		Model 4		Model 5		Model 6	
Publication Type	Adjusted Odds Ratio	*p*	Adjusted Odds Ratio	*p*	Adjusted Odds Ratio	*P*	Adjusted Odds Ratio	*p*	Adjusted Odds Ratio	*p*	Adjusted Odds Ratio	*p*
Panel A. Internist												
Any publication	0.975 [0.911, 1.043]	.458	1.002 [0.941, 1.067]	.945	0.962 [0.897, 1.032]	.282	0.993 [0.931, 1.059]	.832	0.995 [0.934, 1.061]	.884	0.968 [0.902, 1.037]	.354
Average impact factor 3+	1.012 [0.932, 1.098]	.780	1.022 [0.948, 1.102]	.574	1.003 [0.921, 1.093]	.945	1.013 [0.938, 1.095]	.738	1.012 [0.937, 1.093]	.757	1.006 [0.924, 1.095]	.893
Average impact factor 5+	0.958 [0.893, 1.028]	.232	0.976 [0.915, 1.041]	.465	0.942 [0.875, 1.014]	.111	0.963 [0.902, 1.029]	.270	0.967 [0.906, 1.033]	.322	0.948 [0.881, 1.020]	.150
Any clinical study	0.919** [0.849, 0.994]	.036	0.955 [0.885, 1.030]	.231	0.915** [0.844, 0.993]	.033	0.952 [0.881, 1.029]	.215	0.950 [0.880, 1.027]	.196	0.918** [0.847, 0.995]	.037
Average impact factor 3+	0.827*** [0.725, 0.944]	.005	0.865** [0.765, 0.979]	.022	0.816*** [0.712, 0.936]	.004	0.862** [0.760, 0.977]	.021	0.859** [0.760, 0.972]	.016	0.820*** [0.717, 0.937]	.003
Average impact factor 5+	0.791*** [0.690, 0.907]	.001	0.855** [0.747, 0.979]	.024	0.790*** [0.685, 0.911]	.001	0.860** [0.749, 0.988]	.033	0.850** [0.740, 0.975]	.020	0.787*** [0.684, 0.906]	.001
Any basic science study	0.698*** [0.564, 0.864]	.001	0.740*** [0.595, 0.919]	.007	0.695*** [0.563, 0.857]	.001	0.747*** [0.599, 0.931]	.009	0.747*** [0.602, 0.926]	.008	0.700*** [0.571, 0.859]	.001
Average impact factor 3+	1.017 [0.910, 1.136]	.769	1.015 [0.923, 1.116]	.760	1.012 [0.903, 1.134]	.844	1.006 [0.914, 1.108]	.897	1.007 [0.915, 1.108]	.886	1.015 [0.906, 1.136]	.801
Average impact factor 5+	0.948 [0.811, 1.109]	.506	0.968 [0.834, 1.123]	.666	0.929 [0.793, 1.089]	.364	0.951 [0.818, 1.106]	.514	0.951 [0.819, 1.104]	.508	0.935 [0.799, 1.093]	.399
Panel B. Surgeon												
Any publication	1.067 [0.950, 1.198]	.273	1.095* [0.987, 1.215]	.087	1.055 [0.941, 1.182]	.359	1.055 [0.941, 1.182]	.359	1.102* [0.994, 1.221]	.066	1.070 [0.952, 1.203]	.255
Average impact factor 3+	1.000 [0.850, 1.177]	.998	1.030 [0.904, 1.175]	.655	0.988 [0.842, 1.158]	.880	0.988 [0.842, 1.158]	.880	1.024 [0.900, 1.165]	.718	0.995 [0.848, 1.168]	.954
Average impact factor 5+	1.050 [0.945, 1.166]	.364	1.072 [0.972, 1.183]	.165	1.040 [0.936, 1.156]	.460	1.040 [0.936, 1.156]	.460	1.077 [0.977, 1.188]	.136	1.054 [0.952, 1.168]	.309
Any clinical study	1.078 [0.933, 1.247]	.308	1.071 [0.931, 1.232]	.337	1.071 [0.926, 1.239]	.355	1.071 [0.926, 1.239]	.355	1.086 [0.943, 1.251]	.252	1.088 [0.943, 1.256]	.248
Average impact factor 3+	0.869 [0.693, 1.089]	.222	0.910 [0.723, 1.144]	.418	0.846 [0.672, 1.066]	.156	0.846 [0.672, 1.066]	.156	0.915 [0.729, 1.147]	.439	0.874 [0.696, 1.097]	.245
Average impact factor 5+	1.235*** [1.064, 1.433]	.005	1.207** [1.033, 1.410]	.018	1.236*** [1.065, 1.435]	.005	1.236*** [1.065, 1.435]	.005	1.232*** [1.053, 1.441]	.009	1.252*** [1.080, 1.451]	.003
Any basic science study	0.491*** [0.289, 0.834]	.009	0.531** [0.302, 0.933]	.028	0.467*** [0.273, 0.798]	.005	0.467*** [0.273, 0.798]	.005	0.547** [0.313, 0.956]	.034	0.497** [0.291, 0.848]	.010
Average impact factor 3+	0.654*** [0.490, 0.871]	.004	0.702** [0.525, 0.940]	.017	0.640*** [0.478, 0.856]	.003	0.640*** [0.478, 0.856]	.003	0.711** [0.532, 0.949]	.021	0.659*** [0.494, 0.879]	.005
Average impact factor 5+	0.970 [0.778, 1.210]	.789	0.981 [0.775, 1.241]	.872	0.961 [0.771, 1.197]	.720	0.961 [0.771, 1.197]	.720	0.992 [0.787, 1.249]	.943	0.984 [0.792, 1.224]	.888
Patient characteristics	X		X		X		X		X		X	
Hospital fixed effects			X				X				X	
Total fee					X		X					

Patient volume	X	X

Note. Standard deviations are shown in parentheses, and 95% confidence intervals are shown in brackets.
*$p < .10$. **$p < .05$. ***$p < .01$.

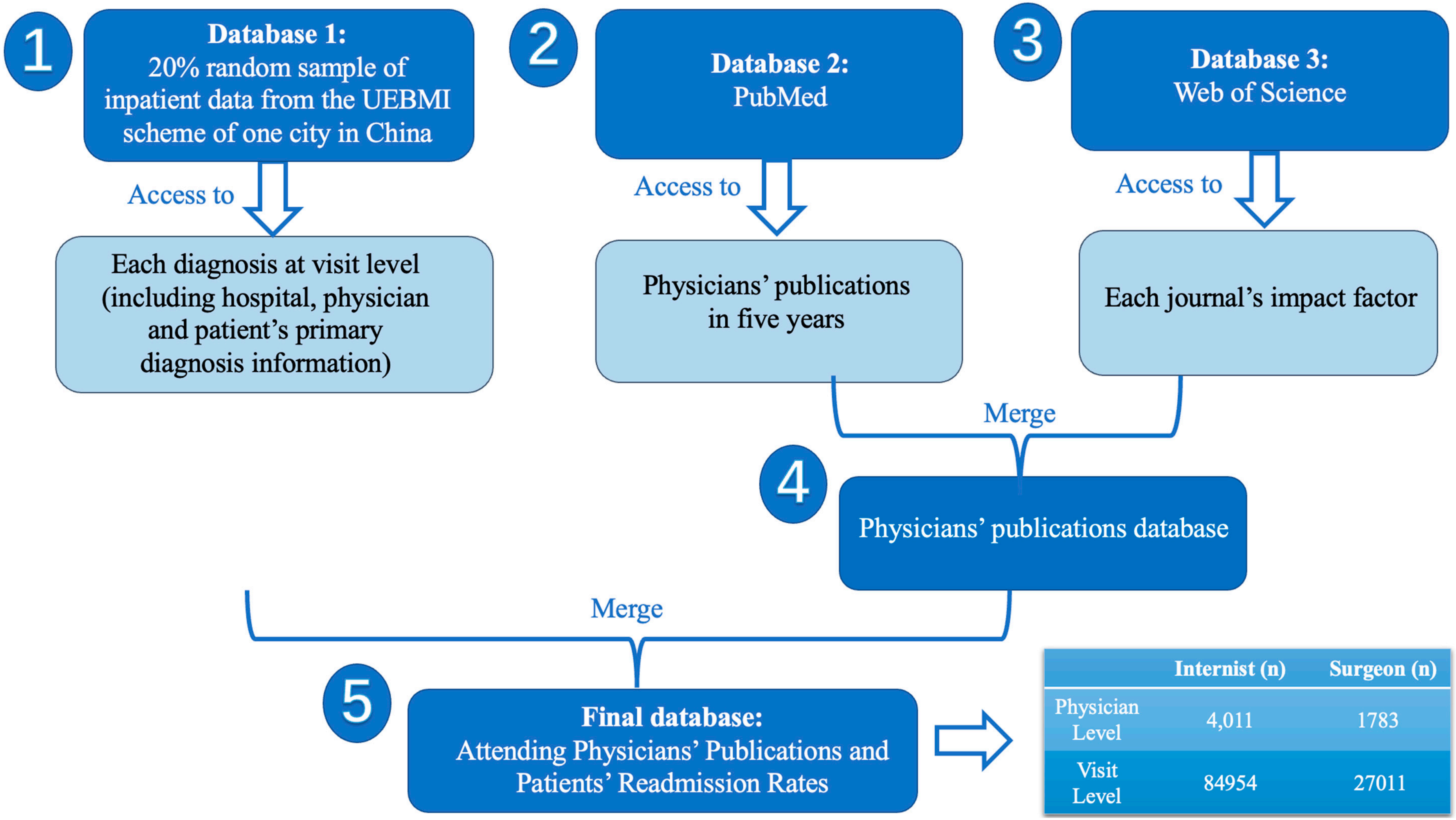

Figure S1. Data linkage process.
